# Relationship between Vitamin D Levels and the Incidence of Post Coronary Artery Bypass Graft Surgery Atrial Fibrillation

**Published:** 2018-10

**Authors:** Maryam Daie, Azita Hajhossein Talasaz, Abbasali Karimi, Kheirollah Gholami, Abbas Salehiomran, Hamid Ariannejad, Arash Jalali

**Affiliations:** 1 Department of Clinical Pharmacy, School of Pharmacy, Tehran University of Medical Sciences, Tehran, Iran.; 2 Tehran Heart Center, Tehran University of Medical Sciences, Tehran, Iran.

**Keywords:** *Atrial fibrillation*, *Vitamin D deficiency*, *Coronary artery bypass*

## Abstract

**Background:** Postoperative atrial fibrillation (POAF) is probably a consequence of inflammation. Vitamin D is known for its anti-inflammatory properties. The aim of this study was to evaluate the effects of vitamin D levels on the incidence of POAF.

**Methods:** In a prospective cohort study, patients were monitored for the occurrence of POAF during the first 5 days after coronary artery bypass grafting surgery in Tehran Heart Center. Those with concomitant valvular surgeries were excluded. Thereafter, they were divided into 2 groups: with or without POAF. Vitamin D levels were assessed in all the patients. The relationship between the vitamin D level and the incidence of POAF was evaluated and compared between the groups using the Mann–Whitney U test.

**Results:** The study population comprised of 156 patients. The mean age was 62.8±8.6 years, and 105 (67.3%) patients were male. Of the 156 patients, 29 (19%) developed POAF. The median preoperative vitamin D level was 15.3 in the group with POAF and 25.3 in the group without POAF (P=0.07).

**Conclusion:** Our results demonstrated no significant relationship between vitamin D levels and the occurrence of POAF.

## Introduction

Postoperative atrial fibrillation (AF) is the most common arrhythmia after coronary artery bypass grafting surgery (CABG).^1^ It usually happens during the first 5 days after surgery, and its incidence has 2 peaks: immediately after surgery and on the second day after surgery. The mechanism of postoperative AF has yet to be fully elucidated, but the most probable mechanism is inflammation. A systematic review concluded that inflammation was the main precipitating factor for the occurrence of AF after cardiac surgery and introduced drugs with anti-inflammatory properties for the prevention of this arrhythmia.^2^ Elevations in inflammation markers such as C-reactive protein (CRP) and interleukin 6, concurrently with postoperative AF, confirm this theory.^3^^, ^^4^ Other suggested mechanisms are neurohormonal activation,^5^ increased sympathetic and parasympathetic activities,^6^ and oxidative stress.^7^

Multiple risk factors have been described for postoperative AF. A preoperative factor is a high score of CHA2DS2-VASc (Congestive heart failure, Hypertension, Age ≥75 y, Diabetes mellitus, prior Stroke or transient ischemic attack or thromboembolism, Vascular disease, Age 65–74 y, Sex category [female sex], obesity, withdrawal of beta-blockers, left atrial enlargement, left ventricular dysfunction, history of AF or other arrhythmias, chronic obstructive pulmonary disease, high cholesterol, chronic kidney disease, intra-aortic balloon pump usage, intraoperative inotrope use, and hypomagnesemia.^6^^, ^^8^^-^^11^ Postoperative AF is less common after isolated CABG than valvular or combined CABG and valvular surgery.^10^^, ^^12^ Some studies have demonstrated a genetic predisposition as a risk factor for postoperative AF.^6^^, ^^13^

The reported incidence rates of postoperative AF are variable and range from 15% to 40%.^1^^, ^^8^ Postoperative AF may cause different major and minor complications such as myocardial infarction, respiratory failure, stroke, decreased short-term and long-term survival, higher costs of treatment, and longer lengths of hospital and intensive care unit (ICU) stay.^11^^, ^^14^^-^^16^ Considering age as the main predictor of postoperative AF and the incremental trend of the age of the patients needing CABG, the incidence of postoperative AF has been on the increase in the last decades.^9^^, ^^17^

Vitamin D is an essential fat-soluble vitamin, whose deficiency is common. Formerly known as a regulatory hormone in calcium and phosphorus metabolism and skeletal health, vitamin D is now noted as an important hormone in such different diseases as skin and autoimmune diseases, cancer, diabetes mellitus, hypertension, and cardiovascular diseases.^18^ Studies have demonstrated a rise in mortality rates in critically ill patients who are deficient in 25-hydroxy vitamin D (25[OH]D). The mentioned patients have longer lengths of stay in the hospital. Sepsis is more common in 25(OH)D-deficient patients.^19^ Epidemiological studies have demonstrated an increase in cardiovascular events in winter, when there is less ultraviolet radiation.^20^ This is interpreted because of a higher prevalence of vitamin D deficiency in winter. Moreover, prior studies have shown the regulatory effects of 25(OH)D on inflammation and the renin–angiotensin system. The relation between vitamin D deficiency and cardiovascular diseases such as hypertension, thrombosis, and coronary heart disease has been established. 

The Endocrine Society’s Clinical Practice Guideline defines vitamin D deficiency as a 25(OH)D level below 20 ng/mL (50 nmol/L) and vitamin D insufficiency as a 25(OH)D level of 21 to 29 ng/mL (52.5–72.5 nmol/L).^21^ CYP27B1 is a receptor expressed by most cardiovascular and inflammatory cells; it enables the local synthesis of 1,25-dihydroxyvitamin D (1,25[OH]_2_D).^22^ This target cell synthesis of 1,25(OH)_2_D seems to be particularly important in the nonskeletal actions of vitamin D. Further, 1,25(OH)_2_D can control inflammatory and immune responses, the main pathogenesis of cardiovascular diseases. The inhibitory effects of vitamin D metabolites on the renin–angiotensin system can explain the role of vitamin D in hypertension. Vitamin D deficiency can activate the renin–angiotensin system and inflammation, leading to a poor prognosis in heart failure, and vitamin D supplementation decreases plasma renin concentration.^23^ While some epidemiological studies have demonstrated the higher prevalence of AF after CABG, some other studies have shown no relationship between vitamin D levels and postoperative AF.^24^^-^^27^ Accordingly, given the controversy surrounding this issue, we sought to evaluate the effects of vitamin D levels on the occurrence of AF after CABG.

## Methods

The study population comprised patients undergoing first-time elective CABG. The patients were at least 18 years old with a normal sinus rhythm and a stable hemodynamic status. Patients with either a known history of AF or a valvular disease were not included. 

The current study was planned as a prospective cohort study in Tehran Heart Center between June 2016 and February 2017. The main aim of this survey was to investigate the prevalence of postoperative AF in isolated CABG patients based on their vitamin D levels. The exclusion criteria consisted of redo surgery; concurrent valvular surgery; valvular diseases or history of valvular surgery; history of any supraventricular arrhythmias; chronic renal failure; chronic hepatic failure; hypercalcemia; hyperthyroidism; hyperparathyroidism; current use of vitamin D supplements or antiepileptics; and use of anti-arrhythmic drugs except for beta-blockers, digoxin, and calcium-channel blockers. Chronic renal failure was defined as a creatinine clearance level of less than 30 mL/min according to the Cockcroft–Gault Equation. 

Patients with the aforementioned conditions were scheduled to have a measurement of their levels of vitamin D, calcium, magnesium, and CRP. Echocardiographic data before surgery were collected. The levels of 25(OH)D were measured in all the patients 48 hours after surgery. The study population was evaluated for the occurrence of postoperative AF for 5 days, as well as serum creatinine elevation and the length of hospital and ICU stay. Additionally, the patients were monitored continuously via 12-lead telemetry 24 hours immediately after surgery until the fifth postoperative day. Postoperative AF was defined as an episode of AF recorded postoperatively on continuous telemetry, and AF was verified using 12-lead electrocardiography. The AF diagnostic criteria, including the absence of discrete P waves, “irregularly irregular” RR intervals, and a range of ventricular rates between 90 and 170 beats/min, were considered for postoperative AF by a cardiologist in our study. Postoperative AF was defined as any postoperative AF episode which lasted more than 5 minutes or needed therapy for hemodynamic instability. The patients were thereafter divided into 2 groups: those with postoperative AF (Group A) and the ones with no postoperative AF (Group B). 

The design of the present study was approved by the institutional ethics committee. A thorough verbal explanation of the study was given to the patients, and written informed consent was obtained from all those accepting to participate in the study. 

Blood samples were drawn from the antecubital vein in the morning before 10:00 am after overnight fasting. Blood was drawn into standardized tubes containing dipotassium ethylenediaminetetraacetic acid (EDTA) and was stored at room temperature. The levels of vitamin D and other biochemical parameters were analyzed in the biochemistry laboratory within 90 minutes of venipuncture.

The statistical analyses were conducted using SPSS for Windows, version 16.0. (Chicago, SPSS Inc). The sustained data are expressed as means±standard deviations (SDs), while the categorical data are presented as percentages. The χ^2 ^test was employed to compare the categorical variables, while the *t *test or the Mann–Whitney *U* test was utilized to compare the parametric and nonparametric constant variables, respectively. Normal distribution was assessed using the Kolmogorov–Smirnov test. A logistic regression analysis was performed to determine the independent predictors of postoperative AF. A P value of less than 0.05 was considered statistically significant. 

The effect size was defined according to post-hoc power analysis. The effect sizes of vitamin d deficiency and insufficiency were calculated. The effect size of vitamin D deficiency and insufficiency was 77.6% and 44.9%, correspondingly. Our results demonstrated that those parameters had a large or very large effect size.

## Results

The study population was comprised of 156 patients: 105 (67.3%) male and 51 (32.7%) female. The patients were between 33 and 88 years old. Among them, 103 (66.0%) patients had vitamin D levels of less than 30 ng/mL, while 53 (34.0%) had vitamin D levels of equal to or more than 30 ng/mL. As is depicted in Table 1, both groups had almost the same age, gender, family history of cardiovascular diseases, dyslipidemia, and hypertension based on the P values. The 2 study groups were also similar with respect to the number of current smokers. The mean ejection fraction was 27.06±4.23% in Group A and 43.34±10.79% in Group B (P=0.306). Table 2 shows that the laboratory and echocardiography data before surgery were similar between Group A and Group B. The mean calcium level after surgery was almost similar in both groups (8.83±2.33 mg/dl in Group A vs. 8.42±0.72 mg/dl in Group B; P=0.400). The mean magnesium level after surgery was the same in the 2 study groups, too (2.17±0.33 meq/l in Group A vs. 2.13±0.35 meq/l in Group B; P=0.684). The mean serum creatinine level after surgery was 1.24±0.68 and 1.15±0.47 in Group A and Group B, respectively (P=0.084). The median CRP level after surgery was 15.36 (IQR_25-75%_: 9.30-18.50) (mg/l) in Group A and 13.50 (IQR_25-75%_: 9.56-19.90) (mg/l) in Group B (P=0.997). The median serum creatinine level after surgery was 1.09 (IQR_25-75%_: 0.98-1.42) mg/dl in Group A and 1.06 (IQR_25-75%_: 0.88-1.29) mg/dl in Group B (P=0.466). The median troponin T level after surgery was 521.55 (IQR_25-75%_: 306.30-757.70) in Group A and 541.40 (IQR_25-75%_: 318.50-807.40) in Group B. The median creatine kinase MB (CK-MB) level was 18.33 (IQR_25-75%_: 8.09-28.49) in Group A and 16.54 (IQR_25-75%_: 10.32-27.60) in Group B (P=0.902). The difference between intraoperative parameters are depicted in table 3.

Of the 156 patients, 29 (19%) patients had postoperative AF during the first 5 days after surgery (Group A). There were 129 (81%) patients in the group without postoperative AF (Group B). The mean preoperative vitamin D level was 22.3 ng/mL in Group A and 27.3 ng/mL in Group B (P=0.070). Group A was comprised of 8 (15.1%) patients whose 25(OH)D levels were sufficient (≥30 ng/mL), 13 (17.8%) patients with insufficient levels of 25(OH)D (≤30 ng/mL), and 8 (26.7%) patients with deficient levels of 25(OH)D (≤15 ng/mL) (P=0.417). Table 4 shows the difference between groups in terms of lab data and medications received in postoperative period.

The median (IQR) length of hospital stay was 10 (IQR_25-75%_: 7.50-12.00) days in Group A and 7 (IQR_25-75%_: 6.00-8.00) days in Group B (P<0.001). The median length of stay in the ICU was 2 (IQR_25-75%_: 2.00-4.50) days in Group A and 2 (IQR_25-75%_: 2.00-3.00) days in Group B (P=0.131).

Among the patients whose 25(OH)D level was more than 30 ng/mL, current smoking was observed in 11 (20.8%) patients while it was observed in 29 (28.2%) patients among the patients whose 25(OH)D was less than 30 ng/mL (P=0.318).

Among the patients whose 25(OH)D level was more than 30 ng/mL, current diabetes mellitus was observed in 16 (55%) patients, while it was observed in 66 (52%) patients among those whose 25(OH)D was less than 30 ng/mL (P=0.0712).

The patients with postoperative AF were treated with amiodarone. The duration of postoperative AF in these patients varied between 1 and 195 hours, but all of them were in normal sinus rhythm when discharged. The highest recorded heart rate was 200 beats/min. There were no patients with stroke or transient ischemic attack, but 1 patient died in Group A in the early postoperative period.

**Table 1:** Comparisons of the baseline characteristics and drug history between Group A (patients with postoperative atrial fibrillation) and Group B (patients with no postoperative atrial fibrillation)*

	Group A (n=29)	Group B (n=127)	P
**Vitamin D (ng/mL) **	15.3 (9.63-32.57)	25.3 (13.75-39.19)	0.070
**Age (y) **	63.89±8.91	61.19±8.90	0.107
**Gender (male) **	18 (62.1)	87 (68.5)	0.505
**Family of CAD**	9 (31.0)	35 (27.6)	0.707
**Dyslipidemia **	13 (44.8)	16 (55.2)	0.685
**MI history **	13 (44.8)	37 (29.1)	0.102
**Hypertension **	17 (58.6)	76 (59.8)	0.904
**Diabetes mellitus **	16 (55.2)	66 (51.9)	0.712
**Current smoker **	8 (27.6)	41 (32.3)	0.873
**BMI (kg/m2 ) **	27.06±4.23	27.29±4.15	0.790
**ACEI/ARB use **	10 (34.5)	52 (40.9)	0.662
**Beta-blocker use **	2 (6.9)	22 (17.3)	0.175
**Statin use **	27 (93.1)	111 (87.4)	0.529

CAD, Coronary artery disease; MI, Myocardial infarction; BMI, Body mass index; ACEI, Angiotensin-converting enzyme inhibitor; ARB, angiotensin receptor blocker

* Data are presented as mean±SD, n (%), or median (IQR_25-75%_).

**Table 2:** Comparisons of the laboratory and echocardiography data before surgery between Group A (patients with postoperative atrial fibrillation) and Group B (patients with no postoperative atrial fibrillation)*

Laboratory Data	Group A (n=29)	Group B (n=127)	P
Magnesium (meq/l)	2.06± 0.21	2.19±0.20	0.028
Calcium (mg/dl)	9.33±0.41	9.43±0.51	0.354
CRP (mg/l)	0.30 (0.08-0.49)	0.28 (0.12-0.78)	0.453
Serum creatinine (mg/dl)	1.00±0.36	1.00±0.28	0.781
Triglycerides (mg/dl)	120.50 (92.50-170.75)	121.0 (97.50-156.00)	0.975
LDL (mg/dl)	74.00 (55.00-94.25)	81.00 (59.50-104.00)	0.341
HDL (mg/dl)	36.77±11.68	35.27±7.81	0.538
Total cholesterol (mg/dl)	139.27±39.38	141.12±35.78	0.815
WBC (WBC/µL)	7935.86±1745.04	8466.39±1987.70	0.256
Hemoglobin (g/dl)	13.42±1.35	13.68±1.72	0.446
TNT	15.19 (11.50-52.37)	14.49 (10.80-24.10)	0.626
CK-MB	1.82 (1.19-2.95)	1.91 (1.36-2.69)	0.623
Ejection fraction (%)	52.70±10.34	54.73±9.32	0.463
Left atrial size (mm)	38.11±3.61	37.04±4.62	0.233
Left ventricular end-systolic size (mm)	32.20±6.71	32.60±7.14	0.325
Left ventricular end-diastolic size (mm)	47.96±6.64	45.60±7.71	0.266

CRP, C-reactive protein; LDL, Low-density lipoprotein; HDL, High-density lipoprotein; WBC, White blood cells; TNT, Troponin T; CK-MB, Creatine kinase MB

* Data are presented as mean±SD, n (%), or median (IQR_25-75%_).

**Table 3:** Comparisons between Group A (patients with postoperative atrial fibrillation) and Group B (patients with no postoperative atrial fibrillation) in terms of intraoperative and postoperative parameters*

Intraoperative and Postoperative Parameters	Group A (n=29)	Group B (n=127)	P
Bypassed vessel count			0.486
1 vessel	4 (13.8)	9 (7.1)	
2 vessels	8 (27.6)	35 (27.6)	
3 vessels	17 (58.6)	83 (65.4)	
Aortic cross-clamp time (min)	40 (29.0)	40 (20.0)	0.498
Length of ICU stay (days)	2 (2.00-4.50)	2 (2.00-3.00)	0.131
Length of hospital stay (days)	10 (7.5-12.00)	7 (6.00-8.00)	< 0.001

* Data are presented as n (%) or median (IQR_25-75%_).

**Table 4:** Comparisons of Group A (patients with postoperative atrial fibrillation) and Group B (patients with no postoperative atrial fibrillation) in terms of postoperative drugs and laboratory data*

Postoperative Drugs and Laboratory Data	Group A (n=29)	Group B (n=127)	P
Beta-blocker	23 (79.3)	104 (81.9)	0.747
Statin	22 (75.9)	107 (84.3)	0.281
Serum creatinine (mg/dl)	1.09 (0.98-1.42)	1.06 (0.88-1.29)	0.466
CRP (mg/l)	15.36 (9.30-18.50)	13.50 (9.56-19.90)	0.997
Calcium (mg/dl)	8.83±2.33	8.40±0.72	0.403
Magnesium (meq/l)	2.17±0.33	2.13±0.35	0.684

* Data are presented as mean±SD, n (%), or median (IQR_25-75%_).

## Discussion

We could not demonstrate any relationship between vitamin D deficiency and postoperative AF, although we observed a strong trend. This may be because there were 53 patients with sufficient levels of 25(OH)D as opposed to 103 patients with deficient levels of 25(OH)D in our study, using a deficiency cutoff level or telemetry, which may miss short and silent AF episodes. In contrast to previous studies, we observed no relationship between 25(OH)D levels and age. The prevalence rate of 25(OH)D deficiency among our patients was 66%. In our study, the mean 25(OH)D level in the postoperative AF group was less than that in the group with no postoperative AF; the difference was, however, nonsignificant. As most of the cardiovascular effects of 25(OH)D are exerted by 1,25(OH)_2_D, this result may be because of the lesser conversion of 25(OH)D to 1,25(OH)_2_D.^24^ The length of hospital stay was shorter in the patients with normal levels of 25(OH)D than in those with levels of less than 30 ng/mL. 

Some earlier studies have shown that 25(OH)D levels are lower among current smokers.^28^ Nonetheless, our results showed no such inverse correlation, which chimes in with the findings of some other previous studies.^29^

Among our patients, diabetes mellitus was more common in the group of patients with vitamin D deficiency; this finding is concordant with the results of some previous investigations.^30^ It should be noted, however, that in contrast to some previous studies, we observed no relationship between hypertension and 25(OH)D levels, which may be because of our small sample size.

Earlier studies have reported conflicting results about the effects of vitamin D on the occurrence of AF and postoperative AF. Experimental studies have shown that vitamin D has anti-arrhythmic effects on atria.

Chen et al.^31^ measured 25(OH)D levels in 162 patients with non-valvular persistent AF and 160 healthy patients who had referred for health screening. The level of vitamin D was significantly lower in the AF group than in the group without AF (18.5±10.3 vs. 21.4±10.7; P<0.05). The authors showed that the incidence of AF in the patients with 25(OH)D levels of less than 20 ng/mL was twice that in the patients with 25(OH)D levels of more than 20 ng/mL. In addition, they reported a negative correlation between 25(OH)D levels and the diameter of the left atrium and high-sensitivity CRP. 

Demir et al.^32^ studied 300 participants in 3 groups of non-valvular AF, valvular AF, and control and concluded that 25(OH)D deficiency was associated with a higher prevalence of valvular AF.

Ozcan et al.^33^ studied the prevalence of new-onset AF in 277 patients with hypertension and showed that the AF incidence increased in the vitamin D-deficient patients. 

In a large cohort study, Zitterman et al.^34^ measured the preoperative level of 25(OH)D in 4418 patients undergoing cardiac surgery and excluded those with heart transplantation and pacemaker/defibrillator implantation as well as those under 18 years of age. The authors investigated major adverse cardiac and cerebrovascular events (MACCEs, comprising in-hospital death, myocardial infarction, low cardiac output syndrome, and stroke, and found the lowest risk of MACCEs in 25(OH)D levels of between 70 and 100 nmol/L. The worst hospital outcome and the highest mortality rate were in 25(OH)D levels of less than 30 nmol/L. 

Skuladottir et al.^25^ measured preoperative and postoperative day 3 levels of 25(OH)D, 25(OH)D_2_, and 25(OH)D_3_ in 118 patients undergoing CABG, valvular surgery or both. The authors recruited patients older than 40 years of age scheduled to undergo elective surgery without a supraventricular arrhythmia or anti-arrhythmic drug use. The prevalence of postoperative AF was as high as 56%, which was probably due to the inclusion of valvular surgeries. Their results showed that the postoperative AF group had a higher 25(OH)D_2 _level, but there was no difference in terms of 25(OH)D and 25(OH)D_3_. 

Emren et al.^26^ studied 283 patients undergoing isolated CABG. Their exclusion criteria consisted of valvular disease, valvular surgery, redo surgery, off-pump surgery, thyroid disease, malignancies, and receiving vitamin D supplements. The authors measured preoperative calcium and 25(OH)D levels and reported that 25% of their patients developed postoperative AF and the level of 25(OH)D was significantly higher in the group without postoperative AF than in the group with postoperative AF. 

Gode et al.^27^ studied 90 isolated CABG patients. Their exclusion criteria comprised redo surgery, valvular preoperative AF, current use of vitamin D supplements or anti-arrhythmic treatment, beating heart surgery, robotic surgery, bleeding revision, chronic renal failure, and hyperthyroidism. The authors reported that the patients who developed AF during the first 5 days (16.6%) had a significantly lower level of 25(OH)D than those who did not.

The salient limitation in the present study is its small sample size; we, therefore, recommend future investigations with larger numbers of patients. Telemetry is liable to miss a large number of short and silent episodes of AF. That we followed up our patients for only as long as their hospital stay is another drawback. In future studies, patients’ long-term survival and complications should be investigated. Moreover, we did not include valvular patients, in whom postoperative AF is more common than in CABG patients. 

## Conclusion

Our results demonstrated no relationship between 25(OH)D levels and postoperative AF. In contrast to some previous studies, we found that higher or lower levels of 25(OH)D were not associated with a significantly different incidence rate of postoperative AF.

## Notes:

***This paper should be cited as: ***Daie M, Talasaz AH, Karimi A, Gholami K, Salehiomran A, Ariannejad H, Jalali A. Relationship between Vitamin D Levels and the Incidence of Postoperative Atrial Fibrillation. J Teh Univ Heart Ctr 2018;13(4):159-165.

## References

[1] Lauer MS, Eagle KA, Buckley MJ, DeSanctis RW (1989). Atrial fibrillation following coronary artery bypass surgery. Prog Cardiovasc Dis.

[2] Jacob KA, Nathoe HM, Dieleman JM, van Osch D, Kluin J, van Dijk D (2014). Inflammation in new-onset atrial fibrillation after cardiac surgery: a systematic review. Eur J Clin Invest.

[3] Li T, Sun ZL, Xie QY (2016). Meta-analysis identifies serum c-reactive protein as an indicator of atrial fibrillation risk after coronary artery bypass graft. Am J Ther.

[4] Gaudino M, Andreotti F, Zamparelli R, Di Castelnuovo A, Nasso G, Burzotta F, Iacoviello L, Donati MB, Schiavello R, Maseri A, Possati G (2003). The -174G/C interleukin-6 polymorphism influences postoperative interleukin-6 levels and postoperative atrial fibrillation. Is atrial fibrillation an inflammatory complication?. Circulation.

[5] Andrews TC, Reimold SC, Berlin JA, Antman EM (1991). Prevention of supraventricular arrhythmias after coronary artery bypass surgery. A meta-analysis of randomized control trials. Circulation.

[6] Echahidi N, Mohty D, Pibarot P, Després JP, O'Hara G, Champagne J, Philippon F, Daleau P, Voisine P, Mathieu P (2007). Obesity and metabolic syndrome are independent risk factors for atrial fibrillation after coronary artery bypass graft surgery. Circulation.

[7] Ramlawi B, Otu H, Mieno S, Boodhwani M, Sodha NR, Clements RT, Bianchi C, Sellke FW (2007). Oxidative stress and atrial fibrillation after cardiac surgery: a case-control study. Ann Thorac Surg.

[8] Kindo M, Minh TH, Gerelli S, Meyer N, Schaeffer M, Perrier S, Bentz J, Announe T, Mommerot A, Collange O, Marguerite S, Thibaud A, Gros H, Billaud P, Mazzucotelli JP (2014). The prothrombotic paradox of severe obesity after cardiac surgery under cardiopulmonary bypass. Thromb Res.

[9] Frendl G, Sodickson AC, Chung MK, Waldo AL, Gersh BJ, Tisdale JE, Calkins H, Aranki S, Kaneko T, Cassivi S, Smith SC Jr, Darbar D, Wee JO, Waddell TK, Amar D, Adler D, American Association for Thoracic Surgery (2014). 2014 AATS guidelines for the prevention and management of perioperative atrial fibrillation and flutter for thoracic surgical procedures. J Thorac Cardiovasc Surg.

[10] Shen J, Lall S, Zheng V, Buckley P, Damiano RJ Jr, Schuessler RB (2011). The persistent problem of new-onset postoperative atrial fibrillation: a single-institution experience over two decades. J Thorac Cardiovasc Surg.

[11] Phan K, Ha HS, Phan S, Medi C, Thomas SP, Yan TD (2015). New-onset atrial fibrillation following coronary bypass surgery predicts long-term mortality: a systematic review and meta-analysis. Eur J Cardiothorac Surg.

[12] Mathew JP, Fontes ML, Tudor IC, Ramsay J, Duke P, Mazer CD, Barash PG, Hsu PH, Mangano DT, Investigators of the Ischemia Research, Education Foundation, Multicenter Study of Perioperative Ischemia Research Group (2004). A multicenter risk index for atrial fibrillation after cardiac surgery. JAMA.

[13] Qu C, Wang XW, Huang C, Qiu F, Xiang XY, Lu ZQ (2015). High mobility group box 1 gene polymorphism is associated with the risk of postoperative atrial fibrillation after coronary artery bypass surgery. J Cardiothorac Surg.

[14] Almassi GH, Schowalter T, Nicolosi AC, Aggarwal A, Moritz TE, Henderson WG, Tarazi R, Shroyer AL, Sethi GK, Grover FL, Hammermeister KE (1997). Atrial fibrillation after cardiac surgery: a major morbid event?. Ann Surg.

[15] Mariscalco G, Engström KG (2009). Postoperative atrial fibrillation is associated with late mortality after coronary surgery, but not after valvular surgery. Ann Thorac Surg.

[16] Hravnak M, Hoffman LA, Saul MI, Zullo TG, Whitman GR (2002). Resource utilization related to atrial fibrillation after coronary artery bypass grafting. Am J Crit Care.

[17] Amar D, Zhang H, Leung DH, Roistacher N, Kadish AH (2002). Older age is the strongest predictor of postoperative atrial fibrillation. Anesthesiology.

[18] Nemerovski CW, Dorsch MP, Simpson RU, Bone HG, Aaronson KD, Bleske BE (2009). Vitamin D and cardiovascular disease. Pharmacotherapy.

[19] Braun A, Chang D, Mahadevappa K, Gibbons FK, Liu Y, Giovannucci E, Christopher KB (2011). Association of low serum 25-hydroxyvitamin D levels and mortality in the critically ill. Crit Care Med.

[20] Scragg R (1981). Seasonality of cardiovascular disease mortality and the possible protective effect of ultra-violet radiation. Int J Epidemiol.

[21] Holick MF, Binkley NC, Bischoff-Ferrari HA, Gordon CM, Hanley DA, Heaney RP, Murad MH, Weaver CM, Endocrine Society (2011). Evaluation, treatment, and prevention of vitamin D deficiency: an Endocrine Society clinical practice guideline. J Clin Endocrinol Metab.

[22] Dickie LJ, Church LD, Coulthard LR, Mathews RJ, Emery P, McDermott MF (2010). Vitamin D3 down-regulates intracellular Toll-like receptor 9 expression and Toll-like receptor 9-induced IL-6 production in human monocytes. Rheumatology (Oxford).

[23] Liu LC, Voors AA, van Veldhuisen DJ, van der Veer E, Belonje AM, Szymanski MK, Silljé HH, van Gilst WH, Jaarsma T, de Boer RA (2011). Vitamin D status and outcomes in heart failure patients. Eur J Heart Fail.

[24] Zittermann A, Kuhn J, Ernst JB, Becker T, Dreier J, Knabbe C, Gummert JF, Börgermann J (2015). 25-hydroxyvitamin D, 1,25-dihydroxyvitamin D and postoperative outcome in cardiac surgery. J Clin Endocrinol Metab.

[25] Skuladottir GV, Cohen A, Arnar DO, Hougaard DM, Torfason B, Palsson R, Indridason OS (2016). Plasma 25-hydroxyvitamin D2 and D3 levels and incidence of postoperative atrial fibrillation. J Nutr Sci.

[26] Emren SV, Aldemir M, Ada F (2016). does deficiency of vitamin d increase new onset atrial fibrillation after coronary artery bypass grafting surgery?. Heart Surg Forum.

[27] Gode S, Aksu T, Demirel A, Sunbul M, Gul M, Bakır I, Yeniterzi M (2016). Effect of vitamin D deficiency on the development of postoperative atrial fibrillation in coronary artery bypass patients. J Cardiovasc Thorac Res.

[28] McCullough ML, Weinstein SJ, Freedman DM, Helzlsouer K, Flanders WD, Koenig K, Kolonel L, Laden F, Le Marchand L, Purdue M, Snyder K, Stevens VL, Stolzenberg-Solomon R, Virtamo J, Yang G, Yu K, Zheng W, Albanes D, Ashby J, Bertrand K, Cai H, Chen Y, Gallicchio L, Giovannucci E, Jacobs EJ, Hankinson SE, Hartge P, Hartmuller V, Harvey C, Hayes RB, Horst RL, Shu XO (2010). Correlates of circulating 25-hydroxyvitamin D: Cohort Consortium Vitamin D Pooling Project of Rarer Cancers. Am J Epidemiol.

[29] van Dam RM, Snijder MB, Dekker JM, Stehouwer CD, Bouter LM, Heine RJ, Lips P (2007). Potentially modifiable determinants of vitamin D status in an older population in the Netherlands: the Hoorn Study. Am J Clin Nutr.

[30] Dong JY, Zhang WG, Chen JJ, Zhang ZL, Han SF, Qin LQ (2013). Vitamin D intake and risk of type 1 diabetes: a meta-analysis of observational studies. Nutrients.

[31] Chen WR, Liu ZY, Shi Y, Yin DW, Wang H, Sha Y, Chen YD (2014). Relation of low vitamin D to nonvalvular persistent atrial fibrillation in Chinese patients. Ann Noninvasive Electrocardiol.

[32] Demir M, Uyan U, Melek M (2014). The effects of vitamin D deficiency on atrial fibrillation. Clin Appl Thromb Hemost.

[33] Ozcan OU, Gurlek A, Gursoy E, Gerede DM, Erol C (2015). Relation of vitamin D deficiency and new-onset atrial fibrillation among hypertensive patients. J Am Soc Hypertens.

[34] Zittermann A, Kuhn J, Dreier J, Knabbe C, Gummert JF, Börgermann J (2013). Vitamin D status and the risk of major adverse cardiac and cerebrovascular events in cardiac surgery. Eur Heart J.

